# Combining MRI and clinical data to detect high relapse risk after the first episode of psychosis

**DOI:** 10.1038/s41537-022-00309-w

**Published:** 2022-11-17

**Authors:** Aleix Solanes, Gisela Mezquida, Joost Janssen, Silvia Amoretti, Antonio Lobo, Ana González-Pinto, Celso Arango, Eduard Vieta, Josefina Castro-Fornieles, Daniel Bergé, Auria Albacete, Eloi Giné, Mara Parellada, Miguel Bernardo, PEPs group (collaborators), Miquel Bioque, Constanza Morén, Laura Pina-Camacho, Covadonga M. Díaz-Caneja, Iñaki Zorrilla, Edurne Garcia Corres, Concepción De-la-Camara, Fe Barcones, María José Escarti, Eduardo Jesus Aguilar, Teresa Legido, Marta Martin, Norma Verdolini, Anabel Martinez-Aran, Immaculada Baeza, Elena de la Serna, Fernando Contreras, Julio Bobes, María Paz García-Portilla, Luis Sanchez-Pastor, Roberto Rodriguez-Jimenez, Judith Usall, Anna Butjosa, Pilar Salgado-Pineda, Raymond Salvador, Edith Pomarol-Clotet, Joaquim Radua

**Affiliations:** 1 Institut d’Investigacions Biomèdiques August Pi i Sunyer (IDIBAPS), Barcelona, Spain; 2 FIDMAG Germanes Hospitalàries Research Foundation, Barcelona, Spain; 3 Department of Psychiatry and Forensic Medicine, Universitat Autònoma de Barcelona, Barcelona, Spain; 4 Barcelona Clínic Schizophrenia Unit, Neuroscience Institute. Hospital Clínic de Barcelona, Barcelona, Spain; 5 Centro de Investigación Biomédica en Red en Salud Mental (CIBERSAM), Instituto de Salud Carlos III, Madrid, Spain; 6 Department of Clinical Foundations, Pharmacology Unit, University of Barcelona, Barcelona, Spain; 7 Department of Child and Adolescent Psychiatry, Institute of Psychiatry Mental Health, Hospital General Universitario Gregorio Marañón, School of Medicine, Universidad Complutense, IiSGM, Madrid, Spain; 8 Department of Medicine, University of Barcelona, Barcelona, Spain; 9 Department of Medicine and Psychiatry, Universidad de Zaragoza, Zaragoza, Spain; 10 Instituto de Investigación Sanitaria Aragón (IIS Aragón), Zaragoza, Spain; 11 Universidad del País Vasco / EHU, Leioa, Bizkaia Spain; 12 Instituto de Investigación Sanitaria Bioaraba, Vitoria-Gasteiz, Alava, Spain; 13 Psychiatric Department, Hospital Universitario de Alava, Vitoria-Gasteiz, Alava, Spain; 14 Barcelona Clínic Bipolar and Depressive Disorders Unit, Institute of Neurosciences, Hospital Clínic de Barcelona, Barcelona, Spain; 15 Department of Child and Adolescent Psychiatry and Psychology, 2017SGR881. Institute of Neuroscience, Hospital Clínic, Barcelona, Spain; 16 Hospital del Mar Medical Research Institute, Barcelona, Spain; 17 Hospital de Mataró, Barcelona, Spain; 18 Department of Psychosis Studies, Institute of Psychiatry, Psychology & Neuroscience, King’s College London, London, United Kingdom; 19 Centre for Psychiatric Research and Education, Department of Clinical Neuroscience, Karolinska Institutet, Stockholm, Sweden; 20 Centro de Investigación Biomédica en Red (CIBER) de Enfermedades Raras (CIBERER), Madrid, Spain; 21 Cellex, IDIBAPS, University of Barcelona-Hospital Clínic of Barcelona, Barcelona, Spain; 22 Hospital Clínico Universitario, Zaragoza, Spain; 23 Servicio Aragonés de la Salud, Centro de Salud de Tarazona, Zaragoza, Spain; 24 Department of Psychiatry, Hospital Clínico Universitario de Valencia, Valencia, Spain; 25 Biomedical Research Institute INCLIVA, Valencia, Spain; 26 Department Psychiatry, Faculty of Medicine, University of Valencia, Valencia, Spain; 27 Psychiatry Unit. Bellvitge University Hospital. IDIBELL, Barcelona, Spain; 28 Department of Psychiatry, Universidad de Oviedo, Oviedo, Spain; 29 Servicio de Salud del Principado de Asturias (SESPA), Oviedo, Spain; 30 Instituto de Investigación Sanitaria del Principado de Asturias (ISPA), Oviedo, Spain; 31 Instituto Universitario de Neurociencias del Principado de Asturias (INEUROPA), Oviedo, Spain; 32 Instituto de Investigación Sanitaria Hospital 12 de Octubre (imas12), Madrid, Spain; 33 CogPsy Group, Universidad Complutense de Madrid (UCM), Madrid, Spain; 34 Parc Sanitari Sant Joan de Déu, Teaching, Research & Innovation Unit, Institut de Recerca Sant Joan de Déu, Barcelona, Spain; 35 Hospital Infanto-juvenil Sant Joan de Déu, Institut de Recerca Sant Joan de Déu, Esplugues de Llobregat, Barcelona, Spain

**Keywords:** Psychosis, Biomarkers, Schizophrenia

## Abstract

Detecting patients at high relapse risk after the first episode of psychosis (HRR-FEP) could help the clinician adjust the preventive treatment. To develop a tool to detect patients at HRR using their baseline clinical and structural MRI, we followed 227 patients with FEP for 18–24 months and applied MRIPredict. We previously optimized the MRI-based machine-learning parameters (combining unmodulated and modulated gray and white matter and using voxel-based ensemble) in two independent datasets. Patients estimated to be at HRR-FEP showed a substantially increased risk of relapse (hazard ratio = 4.58, *P* < 0.05). Accuracy was poorer when we only used clinical or MRI data. We thus show the potential of combining clinical and MRI data to detect which individuals are more likely to relapse, who may benefit from increased frequency of visits, and which are unlikely, who may be currently receiving unnecessary prophylactic treatments. We also provide an updated version of the MRIPredict software.

## Introduction

The discovery of associations between magnetic resonance imaging (MRI) measures and mental disorders^1^ led to an initial enthusiasm about finding MRI-based biomarkers, but we have failed so far. However, new machine-learning methods have reopened the possibility of creating MRI-based tools that, while far from perfect biomarkers, could still help the clinicians^2^. These tools could help the clinicians diagnose, predict the response to treatment, or estimate the risk of a bad outcome, adjusting the overall intervention accordingly.

Up to the moment, most MRI-based machine-learning studies have aimed to classify the individuals (e.g., patient vs. control, or between two diagnoses), and some other research has been devoted to creating models that estimate the risk of a bad outcome. For instance, many studies have investigated whether it is possible to use clinical data^3^, MRI data^4^, or their combination^5^ to detect healthy individuals at high risk for psychosis. These studies have reported higher transition rates to psychosis in individuals that are males, have brief limited intermittent psychotic symptoms, or show reduced cortical gray matter^6,7^.

Conversely, very little research has focused on detecting those patients with first episode of psychosis (FEP) at high relapse risk (HRR). This lack of research is striking because FEP represents one of the main challenges for mental health^8^. Without an appropriate differential diagnosis and early intervention, clinical development after FEP can lead to a chronic condition^9^. Detecting subjects at HRR is crucial since relapse puts their psychosocial recovery at risk, raises the chance of treatment resistance, and has been linked to higher direct and indirect social and economic costs^10^. A few studies have created models to estimate this risk based on clinical data^11,12^, using variables such as the presence of manic and negative symptoms^13–15^, the diagnosis^12,15^, or cannabis use^11,16,17^. Fewer studies have created models to estimate the risk of outcomes other than relapse (e.g., the severity of future symptoms) based on brain MRI data^18,19^, using volumetric brain changes during the first year^20^ or voxel/surface-based data^18^. And to our knowledge, no studies have attempted to create MRI-based relapse risk-estimation models.

This lack of research is unfortunate, given that a structural MRI-based tool able to detect FEP-HRR would be clinically valuable and feasible. It would be valuable because even if the accuracy of the HRR-FEP detection was modest, it could help the clinician adjust the follow-up and treatment of the patients as deemed beneficial^21^. It would be feasible since individuals with a FEP may undergo an MRI to discard organic brain pathology, so that the structural MRI required for this tool would serve both. This better clinical management would reduce the number of relapse-related hospitalizations in patients at HRR-FEP and exclude patients at low relapse risk from therapies unnecessary for them. Therefore, it would improve the quality of life of individuals with a FEP and reduce the burden on National Health System expenditure.

The current study investigated whether structural MRI might help detect patients at HRR-FEP. To this end, we created an HRR-FEP detection tool. Additionally, we report how we previously optimized the MRI-based machine-learning parameters, using two independent datasets to avoid data leakage or over-complexity (see clarification later). We also freely provide the updated MRI-based machine-learning software to allow other groups to develop their own detection models and a website (see “Available resources”) that estimates HRR-FEP quickly to help other groups independently replicate our model’s accuracy assessment.

## Methods

**Fig. 1:**
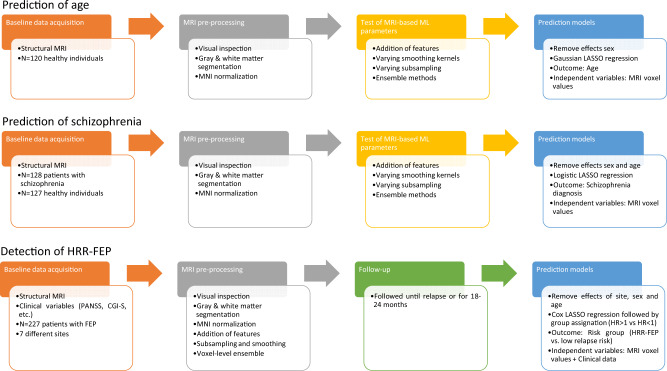
Main study steps. Overall steps followed in this study.

See Fig. 1 for a view of the overall steps of the study. This study complies with the Transparent Reporting of a multivariable prediction model for Individual Prognosis or Diagnosis (TRIPOD, see checklist in the Supplement).

### Participants

The cohort included 227 adolescents/adults with a FEP from 7 different hospitals in Spain, including a previous multicenter study^22,23^, prospectively followed for two years. We invited all patients who met the inclusion criteria during the recruitment periods to join the study. We estimated the sample size based on a previous meta-analysis^24^, in which the relapse rate at two years was around 37%. With this estimation, the overall sample size to detect a hazard ratio (HR) = 2 between patients at HRR-FEP and patients at low relapse risk had to be 190 according to R package powerSurvEpi (https://CRAN.R-project.org/package=powerSurvEpi). We included 20% more to compensate for potential early drop-outs. The mean age was 24.2 years (SD 7.4), and there were 78 females (34.4%) (Table 1). The sample included both young adolescents (12–14 years, *n* = 6) and late adolescents/adults (15–59, *n* = 221); as detailed later, to ensure that the estimation of the model accuracy is not confounded by mixing young adolescents with old adolescents/adults, we repeated the validation of the model after excluding young adolescents.

**Table 1:** Description of the cohort (*n* = 227).

Age	24.2 (7.4)
Sex: female	78 (34.4%)
Familiar psychiatric history	110 (58.2%)
Affective^*^	54 (28.6%)
Suicide^*^	3 (1.4%)
Affective psychosis	48 (21.1%)
Baseline diagnosis	
Schizophrenia	78 (34.4%)
Bipolar disorder	41 (18.1%)
Schizoaffective disorder	12 (5.3%)
Substance-induced psychosis	10 (4.4%)
Major depressive disorder	5 (2.2%)
Other^**^	81 (35.7%)
Positive and Negative Syndrome Scale (PANSS)	71.2 (SD 24.4)
Positive scale	17.6 (SD 7.9)
Negative scale	18.2 (SD 8.5)
General psychopathology scale	35.9 (SD 12.7)
Global Assessment of Functioning scale (GAF)	50.5 (SD 19.7)
Clinical Global Impression scale (CGI-S)	4.4 (SD 1.1)
Young Mania Rating Scale (YMRS)	7.9 (SD 10.2)
Montgomery Asberg Depression rating scale (MADRS)	0.2 (SD 9.9)
Long-acting injectable antipsychotic	15 (6.6%)

^*^Familiar affective history included diagnoses such as bipolar disorder or MDD. Familiar suicide history included consummated attempts.

^**^Other baseline diagnoses included brief psychotic disorder, schizophreniform disorder, delusional disorder, and psychotic disorder not otherwise specified.

Data are presented as mean (SD) or number (%).

We defined relapses as exacerbations of symptoms during at least one week with at least one of eight PANSS items (P1, P2, P3, N1, N4, N6, G5, and G9) scoring above 3 (mild)^25^. On the contrary, remission was defined as scoring <3 in all eight PANSS items. We only considered relapse after at least 6 months of remission.

We detail the inclusion/exclusion criteria and a more detailed description of the cohort in the Supplement. The ethical committees of all hospitals had approved the study, conducted according to the Declaration of Helsinki. Furthermore, all participants and parents/legal guardians for adolescents under 16 had given written informed consent.

### Collection and processing of baseline structural MRI data

We acquired a high-resolution structural image from each participant with a T1-weighted gradient-echo sequence with different devices (see Supplement for details). We used a voxel-based morphometry (VBM) pre-processing pipeline because we have previously found higher accuracy using VBM data^26^ (see Supplement for details).

### Removal of the effects of the site

The effects of the site (e.g., differences in MRI data due to using different devices) might increase noise and confound the analyses. To remove them, we used a recently developed method to control for batch effects named ComBat, as several studies have shown its superiority to simply adding “site” as a covariate in the linear models^27,28^. We found the ComBat parameters (i.e., the MRI differences between sites) using the processed images from exclusively the training set (i.e., we did not use the test set to find the parameters). We then removed the effects of the site from the processed images of both the training and the test sets using these parameters. We must highlight again that the effects of the site were estimated only using individuals from the training set (i.e., not a single piece of information from the test set), thus preventing any information leak. We have previously modified the ComBat functions to allow this separate estimation and application of the ComBat parameters^28^.

We also controlled the effects of the site when estimating the model’s accuracy (see details later), which is important because the effects of the site might bias the accuracy even when researchers attempted to remove them during the creation of the machine-learning model^29^.

### Optimization of MRI-based machine-learning parameters

We optimized the MRI-based machine-learning parameters using two independent datasets. Our main reason for using independent datasets was to avoid any data leakage. We reasoned that if we used the same cohort to optimize the machine-learning parameters and create the risk-estimation model, we could end up validating this model in patients we had previously used to optimize the parameters (based on the best relapse risk estimations). We acknowledge that one strategy to prevent such data leakage would be optimizing the parameters separately for each fold via within-fold cross-validation using the training sets exclusively. However, such a strategy could result in different MRI parameters for the different folds, creating over-complexity in the model. Rather, we looked for general MRI settings that would be stable not only for the different folds but for different predictions or studies.

One dataset included 120 healthy individuals^30^, and we used their MRI data to predict a continuous variable (their age). The other dataset included 255 individuals, half of them with a schizophrenia diagnosis^26,30,31^, and we used their MRI data to predict a binary variable (whether they had received the schizophrenia diagnosis or not). See the Supplement for details. The creation of machine-learning models was analog to the one described later.

We defined the default settings as unmodulated gray matter images, smoothed with a kernel of σ = 4 mm (corresponding to FWHM = 9.5 mm) and a voxel size of 3 × 3 × 3 mm^3^. We tested whether the accuracy of MRI-based machine-learning models depended on: the addition of features (gray and white matter images, modulated and unmodulated images as they convey complementary volumetric information^30^, global gray matter volume and global brain volume, and the midline abnormalities cavum septum pellucidum and absence of adhesion interthalamica, previously reported as good predictors in FEP^31,32^), the size of the smoothing kernels (from σ = 2 to 6 mm, corresponding to FWHM ≈ 5.3–15.8 mm, i.e., encompassing the usual widths of standard neuroimaging software) since the previous literature differs in the optimal kernel size^18,26^, or the use of ensemble methods. Ensemble learning methods seek better prediction performance and robustness by combining the predictions of different models. We used two ensemble methods: (a) we resampled the subjects with replacement 18 times and repeated the creation of the risk-estimation model with each of the 18 resampled datasets, and (b) we selected half of the brain 18 times (i.e., dividing the brain in different angles) and repeated the creation of the risk-estimation model with each of the 18 half brains. Any of these two ensemble methods resulted in 18 risk-estimation models, which we applied to the test set, resulting in 18 risk estimations per patient. Finally, we calculated the mean of the 18 risk estimations to obtain a single risk-estimation per patient.

On another note, we tested two approaches to reduce the computational cost: applying additional subsampling (6 × 6 × 6 mm^3^ or 12 × 12 × 12 mm^3^, instead of 3 × 3 × 3 mm^3^) and limiting the analyses to statistically significant voxels (*P* < 0.05 uncorrected at the univariate analysis).

We defined the accuracy of the age predictions as the mean absolute error (MAE) between the predicted and the actual age and the accuracy of the diagnostic predictions as the proportion of correct predictions. Finally, we assessed whether differences in accuracy between the analysis using a given parameter and the reference analysis (unmodulated gray matter smoothed with σ = 4 mm) were statistically significant by conducting a paired-sample Wilcoxon test of the absolute errors of the two analyses.

### Creation and validation of the HRR-FEP detection tool

**Fig. 2:**
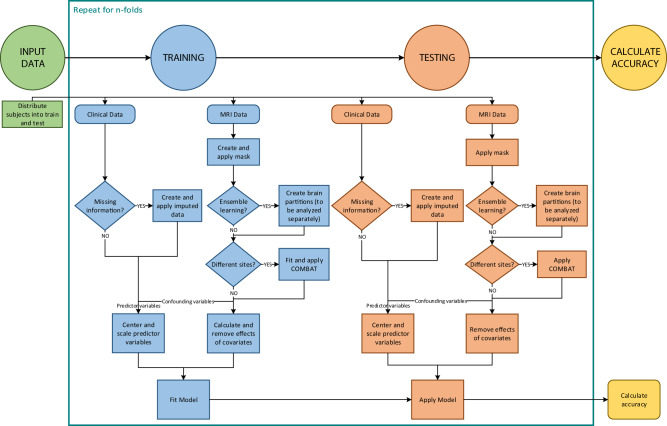
MRIPredict flowchart. Creation of the high relapse risk after the first episode of psychosis (HRR-FEP) detection tool.

We used a cross-validation scheme to create the tool using a set of patients and validate it using a new set of patients. Specifically, we randomly divided the overall cohort into ten groups or “folds” trying to preserve a similar number of relapses in each fold. First, we created the model using data from individuals from folds 2 to 10 (the “training set”), and we estimated the relapse risk of individuals from fold 1 (the “test set”) (Fig. 2). We then created the model using data from individuals from folds 1 and 3–10, and we estimated the relapse risk of individuals from fold 2. And so on. Therefore, we could estimate the relapse risk of all individuals, but we never used the same individuals for training and validating the model.

The creation of the HRR-FEP models in the training set consisted of fitting a multiple regression. The dependent variable was the time to relapse. The independent variables were the clinical data (including the items from the symptom scales PANSS, GAF, MADRS, YMRS, the diagnosis, and whether the patient was taking long-acting injectable antipsychotic treatment) and the voxel values of the pre-processed MRI. Before conducting the regression, we removed the effects of age and sex from the training MRI data with standard linear models. We must highlight once more that the effects of age and sex were estimated only using individuals from the training set (i.e., not a single information from the test set), thus preventing any information leak. We also scaled the clinical variables to the [0-1] range to have a distribution like the MRI voxels. To avoid overfitting, we used a “lasso” regression, which automatically selects a few regressors by penalizing the sum of the absolute value of the coefficients and has been proven to be able to deal with high-dimensional data and still achieve high-performance models^33^. A regularization parameter defines the amount of penalization, ranging from null (no penalization, as in a standard regression) to infinity (maximum penalization). This regularization parameter is automatedly selected by the algorithm via internal cross-validation within the training set. We chose the lasso regression algorithm for its good performance^26^, simplicity, and adequacy for survival analyses. All these previous steps estimated using the training set were applied later to the test set to validate the performance of the model.

In other words, we found a risk-estimation model using the patients of the training set exclusively, and afterward, we applied the model to the patients of the test set to estimate their risk of relapse. To estimate a patient’s risk, we multiplied each coefficient of the lasso model (see Table 3) by the value of the variable in the patient and added the results. If the sum was >0 (corresponding to a HR > 1), we considered that the patient was at HRR-FEP. Conversely, if the sum was ≤0 (corresponding to HR ≤ 1), we considered the patient at low relapse risk.

To test whether individuals estimated to be at HRR-FEP had statistically more relapses than individuals at low relapse risk, we used the “multisite.accuracy” package^29^, which considers the site’s residual effects when estimating the accuracy. Specifically, we conducted a mixed-effects Cox proportional hazards regression (https://CRAN.R-project.org/package=coxme). The dependent variable was the time to relapse. The independent variable was the estimated risk group (HRR-FEP vs. low relapse risk), and the site was a random factor of no interest.

To rule out whether the model’s accuracy could mainly depend on MRI data or clinical data, we also created HRR-FEP detection tools exclusively based on MRI data or clinical data. Also, for descriptive purposes, we mapped the brain regions univariately associated with increased or decreased relapse risk after the FEP using standard survival analyses (see Supplement).

We conducted the analyses with our freely available graphical software MRIPredict (which we have updated for this work), based on the “glmnet” package for R (https://glmnet.stanford.edu/).

### Available resources

Groups interested in conducting similar analyses can download our free graphical-user-interface MRIPredict software at https://www.mripredict.com/.

We encourage independent groups to replicate our model’s accuracy assessment. To help them, we provide a website-based version of the tool (https://www.mripredict.com/hrr-fep/) that quickly estimates the HRR-FEP of an individual. For the website, we fitted a model using the whole cohort and selected the coefficients with an absolute value ≥0.05 (see Supplement); its risk estimations seem perfect (all relapses are in HRR-FEP individuals). However, this accuracy is inflated because it uses the same individuals for training and testing; we obtained a more reliable accuracy estimation with cross-validation (see next). In addition, we only offer this tool to support replication by other researchers; the tool estimations must be considered experimental.

## Results

### Cohort description

There were 16 relapses, representing a 9.4% relapse rate at 24 months. Note that while the number of relapses was limited, it still yielded enough statistical power to detect meaningful differences in relapse risk between groups (e.g., using the R package powerSurvEpi, we estimated that we had 70%/80%/90% power to detect a HR = 4.3/5.7/9.5). The median time from scan to relapse (in patients who had a relapse during the follow-up) was 7.4 months, and the median time from scan to the last follow-up visit (in patients with no relapse during the follow-up) was 23.7 months. We detected no statistically significant differences in relapse risk between affective and non-affective psychosis or between diagnoses, except increased risk in patients with a schizoaffective disorder diagnosis (HR = 3.6, *P* = 0.046).

### Optimal MRI-based machine-learning parameters

When optimizing the MRI-based machine-learning parameters, we found that adding gray and white matter images, unmodulated modulated images, and the use of a voxel-level ensemble improved the accuracy (Table 2). Conversely, using subject-level ensemble worsened the accuracy. The other varying parameters did not influence accuracy. We thus selected the addition of gray and white matter images, unmodulated and modulated images, and the use of a voxel-level ensemble for the HRR-FEP analyses. We also chose triple subsampling because it makes all calculations substantially less computationally expensive.

**Table 2:** Optimization of MRI-based machine-learning parameters.

Adjustment		Age predictions			Diagnostic predictions		
		MAE	Absolute *P* value^(a)^	Relative *P* value^(b)^	Accuracy	Absolute *P* value^(a)^	Relative *P* value^(b)^
Addition of features	No (reference)	7.4 years	<0.001	–	70.8%	<0.001	–
	+ white matter and modulated images	6.2 years	<0.001	0.006	68.6%	<0.001	0.039
	+ global volumes	7.4 years	<0.001	n.s.	70.8%	<0.001	n.s.
	+ midline abnormalities	–	–	–	70.7%	<0.001	n.s.
						<0.001	
Varying smoothing kernel width	σ = 2 mm	7.8 years	<0.001	n.s.	68.9%	<0.001	n.s.
	σ = 3 mm	7.2 years	<0.001	n.s.	69.4%	<0.001	n.s.
	σ = 4 mm (reference)	7.4 years	<0.001	–	70.8%	<0.001	–
	σ = 5 mm	7.5 years	<0.001	n.s.	71.2%	<0.001	n.s.
	σ = 6 mm	7.5 years	<0.001	n.s.	70.8%	<0.001	n.s.
						<0.001	
Subsampling	Single (reference)	7.4 years	<0.001	–	70.8%	<0.001	–
	Double subsampling	7.5 years	<0.001	n.s.	70.6%	<0.001	n.s.
	Triple subsampling	7.3 years	<0.001	n.s.	70.3%	<0.001	n.s.
	Only statistically significant	7.4 years	<0.001	n.s.	71.0%	<0.001	n.s.
						<0.001	
Ensemble	No (reference)	7.4 years	<0.001	–	70.8%	<0.001	–
	Subjects	8.5 years	<0.001	<0.001	64.4%	<0.001	0.001
	Voxels (half brains)	7.1 years	<0.001	0.002	73.2%	<0.001	0.034
						<0.001	
Optimal parameters	+ white matter and modulated images, triple subsampling, the ensemble of voxels	6.3 years	<0.001	<0.001	74.4%	<0.001	0.043

*MAE* mean absolute error.

(a) Wilcoxon test comparing the predictions obtained with these settings with the predictions obtained with a null model (i.e., predicting that all individuals have the average age of the sample for age predictions, or to flipping a coin for diagnostic predictions).

(b) Wilcoxon test comparing the predictions obtained with these setting with the predictions obtained with the reference settings (unmodulated gray matter smoothed with σ = 4 mm and no subsampling or ensemble).

### HRR-FEP detection

**Fig. 3:**
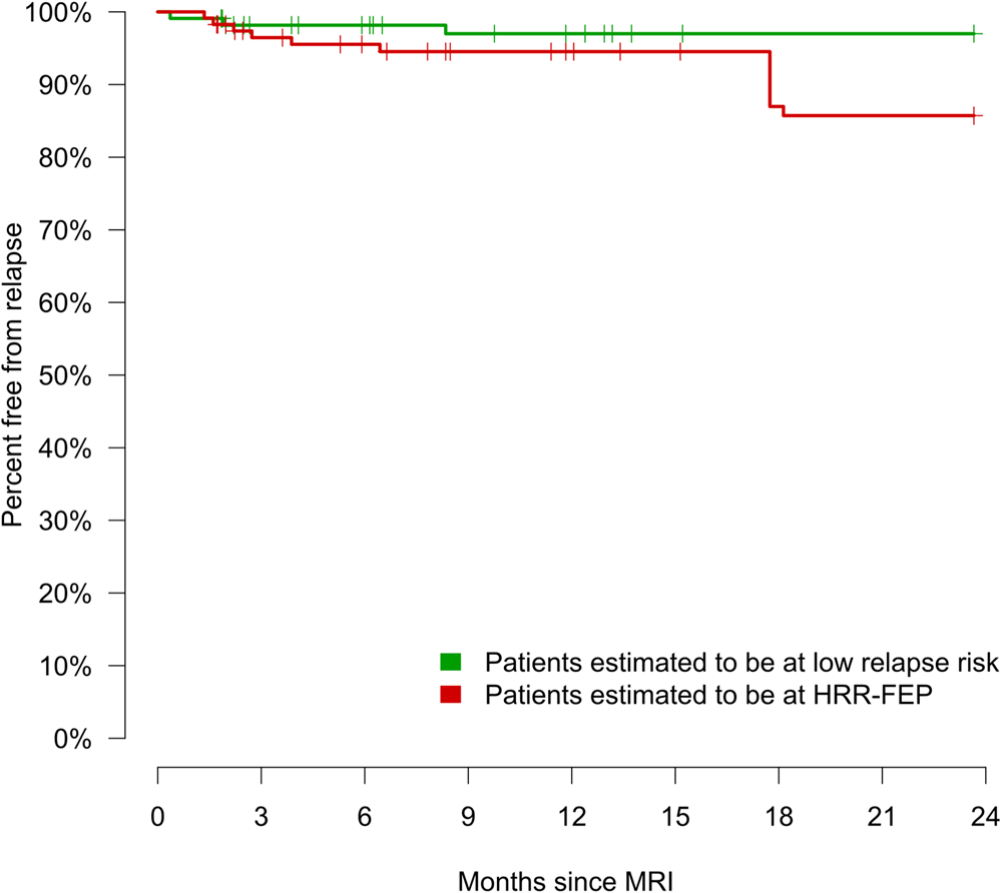
Observed relapses depending on estimated risk group. Kaplan–Meier curves of the observed relapse in patients estimated to be at high relapse risk after the first episode of psychosis (HRR-FEP) vs. patients at low relapse risk.

The Cox regression of the time to relapse comparing patients estimated to be at HRR-FEP vs. low relapse risk was clinically relevant (HR = 4.58, i.e., HRR-FEP patients had five times more risk to relapse) and had a (borderline) statistical significance (HR 95% confidence interval = 1.01–20.74, Z = 1.98, *P* = 0.048, Fig. 3). The results were identical when we excluded young adolescents (i.e., 12–14-years-old). In the 114 individuals estimated to be at HRR-FEP, there were 13 relapses, representing a 14.8% relapse rate at 24 months. Conversely, there were only three relapses in the 113 individuals estimated to not be at HRR-FEP, representing a 2.9% relapse rate at 24 months. Using the R package powerSurvEpi, we estimated that the power to detect a HR = 4.58 with 16 relapses is 72%. The variables automatedly selected by the lasso regression to create the HRR-FEP detection tool were the diagnosis of schizoaffective disorder, the lack of difficulty in abstract thinking and poor impulse control, and the increase or decrease of unmodulated and modulated gray and white matter in several brain regions. Table 3 details the specific brain regions and clinical variables detected in the descriptive univariate analysis and the machine-learning model. We report the entire machine-learning model in the Supplement.

**Table 3:** Descriptive univariate analysis and machine-learning estimators of high relapse risk after the first episode of psychosis (HRR-FEP).

	Descriptive univariate analysis	Machine learning
Clinical variables		
Schizoaffective disorder	HR = 3.6, *P* = 0.046	β = +0.24
↓ Poor rapport (PANSS N3)	–	β = −0.01
↓ Difficulty in abstract thinking (PANSS N5)	HR = 0.6, *P* = 0.044	β = −0.074
↓ Conceptual disorganization (PANSS P2)	–	β = −0.01
↓ Poor attention (PANSS G11)	–	β = −0.04
↑ Age in years	HR = 1.1, *P* = 0.008	–
↑ Long-acting injectable antipsychotic	–	β = 0.01
Gray matter increase		
↑ R Postcentral	–	Unm, [54, −6, 24], β = +0.93
Gray matter decrease		
↓ R middle temporal	–	Unm, [66, −6, −12], β = −0.43
↓ R inferior frontal/precentral	–	Unm, [30, 6, 36], β = −0.21 Mod, [42, 6, 36], β = −0.18
↓ R middle frontal	–	Unm, [30, 42, 36], β = −0.20
↓ R/L rectus	Unm, [−6, 30, −36], z = −2.6	Mod, [6, 30, −24], β = −0.17 Unm, [6, 30, −24], β = −0.15
↓ L superior frontal	Unm, [−18, 66, −24], z = −2.8	–
↓ R medial frontal	Unm, [6, 78, −12], z = −2.6	–
↓ R Angular	–	Unm, [30, −54, 36], β = −0.05
White matter increase		
↑ R precentral	–	Unm, [42, 6, 36], β = +0.54
↑ L Middle frontal	–	Unm, [−42, 6, 36], β = +0.10
White matter decrease		
↓ R middle frontal	–	Unm, [30, 30, 36], β = -0.86
↓ L inferior frontal	–	Mod, [−42, 18, 12], β = −0.73 Unm, [−42, 18, 12], β = −0.57
↓ R Cuneus	–	Mod, [18, −90, 12], β = −0.18
↓ R superior frontal	Unm, [6, 54, 24], z = 2.9	–
↓ L corpus callosum	Unm, [−18, 18, 24], z = 2.7	Unm, [−18, −30, 24], β = −0.05
↓ R corpus callosum	–	Mod, [6, 30, 0], β = −0.05
↓ L Middle frontal	–	Mod, [−30, 42, 12], β = −0.06
↓ R postcentral	–	Mod, [54, −6, 24], β = −0.09

*L* left, *Mod* modulate, *PANSS* Positive and Negative Syndrome Scale, *R* right, *Unm* unmodulated.

In the descriptive univariate analysis, we only report the peaks of MRI clusters with voxel uncorrected *P* value <0.005 and the clinical variables with uncorrected *P* value <0.05. For the sake of simplicity, we only report here the machine-learning coefficients with an absolute value ≥0.01 for clinical variables and ≥0.05 for MRI voxels; see the entire model in the Supplement.

The HRR-FEP detection tools exclusively using MRI data or solely based on clinical variables failed to detect patients at HRR-FEP.

## Discussion

In this work, we created an MRI-based machine-learning tool to detect those patients at HRR-FEP using a cohort of 227 individuals with a FEP. The model showed to detect HRR-FEP successfully. The hazard of relapse was 4.5 larger in individuals estimated to be at HRR-FEP than in low relapse risk individuals (14.8% vs. 2.9% relapse rate at 2 years), and we estimated the power to detect such a hazard ratio of 4.5 with 16 relapses is 72%.

The study thus achieved the aim of creating a tool that may provide valuable information to the mental health professional. Ideally, the clinician could input the tool with a few MRI and clinical data to know if the patient is estimated to be at HRR-FEP or not, and thus adjust the prophylactic treatment. Knowing this information early is important because currently, clinicians can only know which patients are at HRR-FEP after several relapses. And before that, patients at HRR-FEP may experience repeated relapses if the prevention is too weak, while patients at low relapse risk may experience increased adverse events if the prevention is too strong. That said, any adjustment of the prophylactic treatment should follow the “first, do no harm” principle because in our cohort, most (85%) individuals estimated to be at HRR-FEP did indeed not relapse. Not less important, the clinician could also consider removing or reducing the prophylactic treatment in individuals estimated to be unlikely to relapse. These patients currently may be receiving treatments that, if the patient is truly unlikely to relapse, may be little useful while harmful.

However, in any case, we want to highlight the need to validate the HRR-FEP detection tool before recommending it. We have noted previously that independent studies often fail to replicate the accuracy reported in mental health machine-learning publications^34^, and our study may not be an exception. We cannot share participant data for privacy reasons. However, we provide the trained classifiers online so that independent researchers can still try to replicate our study results. This approach has been stated to be one of the most convincing forms of replication^35^. However, without intending to create hype, we also think that our work shows the potential clinical utility of MRI-based machine-learning when understood as a source of additional information for the psychiatrist.

We also want to highlight that this tool could be complemented by other tools that update the relapse risk during the follow-up. For example, we have reported for other disorders that the relapse risk at 12 months substantially decreases in patients who have been relapse-free for at least one year^24^. Thus, some patients initially at HRR-FEP may later be at low relapse risk. Similarly, information about changes in the first months could also likely offer valuable information for updating the risk estimation^20^. In this context, we would like to note that, as far as relapses also depend on events that will happen during the follow-up, it is unlikely that a machine-learning model that only uses baseline data scan achieves high risk-estimation accuracy.

A particularity of our study is that, instead of focusing on detecting those healthy individuals at high risk for FEP, it focuses on detecting those FEP patients at HRR. Many studies have already been published regarding predicting transition to psychosis, with varied results^3,4^. Conversely, no studies have been conducted to estimate HRR-FEP from MRI data to our knowledge. This lack of research is striking because assessing the relapse risk is essential to properly adjusting the preventive antipsychotic dose.

Our tool requires an MRI, but patients with a FEP may indeed already undergo an MRI to discard organic brain pathology, so that the structural MRI required for our tool would serve both. This fact increases the feasibility of the HRR-FEP detection tool, given that for many patients, it would only involve minor calculations on any computer. The context is different, for instance, for the detection of individuals with a higher risk of psychosis in the general population, where screening detection tools should only require inputting a small amount of available information. An example of such a screening detection tool is the Psychosis Polyrisc Score (PSS)^36^, which only asks about the presence of a few risk factors^37^ and has shown feasible in a real-world digital implementation^38^.

Interestingly, the accuracy of HRR-FEP detection tools was poorer when we created machine-learning models that used only clinical data or only MRI data. Ad hoc, it may seem evident that the more information, the better the detection. However, many previous studies only used MRI to find biomarkers that should surpass clinical judgment. These may include serum component protein 4 (C4)^39^, polygenic related Risk Score (PRS)^40^, neuroanatomical variables^18,20^. Thus, poetically, we have found that, in the fight between clinical-based and biomarker-based psychiatry, joining efforts predicts better.

One key variable selected by the lasso regression was the diagnosis of schizoaffective disorder; this partly agrees with previous studies reporting associations of diagnosis or manic symptoms with increased relapse rate^12,13,15^. In addition, we think that in the current debate about the validity of DSM/ICD diagnoses, it is worth noting that diagnostic labels more than clinical scales helped predict future relapses. That said, this debate is entirely out of the scope of this paper. On another note, the protective effects of the difficulty in abstract thinking and poor impulse control are intriguing. We speculate that these symptoms may be related to latent disorder subtypes that might be clearer in subsequent phases of the illness. Finally, we must acknowledge that the variables showing statistical significance in the descriptive univariate analysis (see Supplement) were primarily different from the variables selected by the lasso regression. However, this disagreement is expectable because the latter only aims to predict and thus discards brain regions that do not add much to the prediction accuracy, even if they are statistically significant when considered alone.

Before creating the HRR-FEP detection tool, we used two independent datasets to find the optimal parameters for VBM-based machine learning. Finding the optimal parameters in two different datasets keeps the main study data unseen until we create the model for the HRR -FEP detection tool. We acknowledge that the accuracy of the age predictions was lower than that reported elsewhere^41^. This lower accuracy was probably related to the limited sample size of the age prediction dataset. However, we only aimed to compare the accuracy depending on different parameters. We found that the optimal parameters were the addition of gray and white matter images, the addition of unmodulated and modulated images, and the use of voxel-level ensemble. We encourage future studies to use these parameters. Also, we found that even triple subsampling did not affect the accuracy while substantially reducing computational costs.

We want to comment that, while previous work has searched for gold biomarkers with little success, this work shows the potential clinical use of MRI-based machine learning in risk assessment. We speculate that such risk assessment will very likely be far from perfect, i.e., we will not be able to know for sure which patients will have a relapse and which will not, or the date of the relapse. Indeed, such predictions may seem unrealistic considering that relapses also depend on life events and stressors after the assessment^42^. However, the estimation will be clinically valuable as far as we can estimate risk with enough accuracy to help the physician, i.e., so that the information translates into in an effective improvement of the care. Our study does not provide this level of accuracy yet, but we hope to have made a step for future studies.

This work has some limitations. First, this sample does not include the patients who did not meet the inclusion criteria or refused to participate in the study, who may differ from those included. It is a common limitation in many other studies. Second, even if we included 227 patients and followed them for 18–24 months, representing one of the largest brain imaging FEP cohorts worldwide, there were only 16 relapses. This relapse rate is lower than those reported in some previous cohorts^24,43,44^. To check whether the difference in relapse rate was due to our relapse criteria being only based on PANSS while others also considered hospitalizations, we retrieved hospitalizations, and the updated relapse rate (37%) was more in agreement with previous cohorts. However, we could not successfully repeat the analyses with hospitalizations because this information was unavailable on some sites. Third, the statistical significance was weak, probably due to our cohort’s limited number of relapses. In any case, the power to detect a hazard of relapse of 4.5 with the sample size and the number of relapses in this study was 72%, very close to the conventional 80% required in sample size calculations. Fourth, more complex machine-learning algorithms, such as neural networks, might detect more patterns than the relatively simple algorithms used here. However, these algorithms usually require substantially larger cohorts, which may be challenging to achieve. Fifth, for simplicity, we considered patients estimated to have a HR > 1 at HRR-FEP. However, the optimal division between groups could be at another HR threshold. Future studies evaluating the benefits and costs of the interventions at different HR levels may provide more insights into this question. Sixth, we could not evaluate medication adherence, DUP, and premorbid functioning because data was missing in some sites. Due to its established role in relapse, the use of these variables could improve model accuracy. Finally, we could not report statistics such as sensitivity and specificity. We could not estimate such statistics because our data was not binary (relapse vs. not relapse). Note that 38% of patients did not complete the follow-up, and thus we could not classify them as relapse or not relapse - we knew that they had not relapsed until the last visit, but we did not know if they had relapsed afterward. However, even if there were no follow-up losses, we would still report the Cox regression as the primary validation statistic because it considers whether relapses occurred earlier or later. In contrast, binary statistics do not.

To conclude, this study might represent a step towards a translational application of neuroimaging to mental health. Up to now, brain imaging prediction models have mainly aimed to imitate clinical judgment, for example, by training a support vector machine to differentiate between patients and controls based on their brain images^45^. Conversely, we combined clinical and MRI data to improve the accuracy of a tool that, instead of finding reliable biomarkers, aims to help the clinician, ultimately paving the way toward more personalized medicine in mental disorders.

## Supplementary information

Supplement material

## Data Availability

Data are available upon request to the Research Ethics Committees of Benito Menni CASM, Hospital General de Granollers, Hospital de Mataró, Hospital Sant Rafael, Hospital de Bellvitge, Hospital Clínic de Barcelona, Hospital Universitario 12 de Octubre, Hospital Clínic de València, Hospital del Mar, Instituto de Investigación Sanitaria Aragón, Hospital General Universitario Gregorio Marañón, Hospital Sant Joan de Déu Barcelona, Hospital Santiago Apóstol de Vitoria-Gasteiz, and Servicio de Salud del Principado de Asturias.

## Code availability

Groups interested in conducting similar analyses can download and check the R code of the software used to conduct the analysis of this study, and use our free graphical-user-interface MRIPredict software at https://www.mripredict.com/.

## References

[1] DeLisi LE (1985). Cerebral ventricular enlargement as a possible genetic marker for schizophrenia. Psychopharmacol. Bull..

[2] Radua J, Carvalho AF (2021). Route map for machine learning in psychiatry: absence of bias, reproducibility, and utility. Eur. Neuropsychopharmacol..

[3] Rosen M (2021). Towards clinical application of prediction models for transition to psychosis: a systematic review and external validation study in the PRONIA sample. Neurosci. Biobehav. Rev..

[4] Smieskova R (2010). Neuroimaging predictors of transition to psychosis-a systematic review and meta-analysis. Neurosci. Biobehav. Rev..

[5] Schmidt A (2017). Improving prognostic accuracy in subjects at clinical high risk for psychosis: systematic review of predictive models and meta-analytical sequential testing simulation. Schizophrenia Bull..

[6] Salazar de Pablo, G. et al. Probability of transition to psychosis in individuals at clinical high risk: an updated meta-analysis. *JAMA Psychiatry*10.1001/jamapsychiatry.2021.0830 (2021). 10.1001/jamapsychiatry.2021.0830 PMC8281006 34259821

[7] Fortea A (2021). Cortical gray matter reduction precedes transition to psychosis in individuals at clinical high-risk for psychosis: a voxel-based meta-analysis. Schizophrenia Res..

[8] Harrison G (2001). Recovery from psychotic illness: a 15- and 25-year international follow-up study. Br. J. Psychiatry.

[9] Bernardo M (2021). The prevention of relapses in first episodes of schizophrenia: the 2EPs Project, background, rationale and study design. Revista de psiquiatria y salud mental.

[10] Ascher-Svanum H (2010). The cost of relapse and the predictors of relapse in the treatment of schizophrenia. BMC Psychiatry.

[11] Bhattacharyya S (2021). Individualized prediction of 2-year risk of relapse as indexed by psychiatric hospitalization following psychosis onset: model development in two first episode samples. Schizophrenia Res..

[12] Puntis S, Whiting D, Pappa S, Lennox B (2021). Development and external validation of an admission risk prediction model after treatment from early intervention in psychosis services. Transl. Psychiatry.

[13] Arrasate, M. et al. Prognostic value of affective symptoms in first-admission psychotic patients. *Int. J. Mol. Sci.* **17**, 10.3390/ijms17071039 (2016). 10.3390/ijms17071039 PMC4964415 27376266

[14] Wunderink L (2020). Negative symptoms predict high relapse rates and both predict less favorable functional outcome in first episode psychosis, independent of treatment strategy. Schizophrenia Res..

[15] Hui CL (2019). Predicting first-episode psychosis patients who will never relapse over 10 years. Psychological Med..

[16] Berge D (2016). Predictors of relapse and functioning in first-episode psychosis: a two-year follow-up study. Psychiatric Services.

[17] Schoeler T (2017). Poor medication adherence and risk of relapse associated with continued cannabis use in patients with first-episode psychosis: a prospective analysis. Lancet. Psychiatry.

[18] Nieuwenhuis M (2017). Multi-center MRI prediction models: predicting sex and illness course in first episode psychosis patients. NeuroImage.

[19] Dazzan P (2018). Clinical utility of MRI scanning in first episode psychosis. Schizophrenia Bull..

[20] Cahn W (2006). Brain volume changes in the first year of illness and 5-year outcome of schizophrenia. Br. J. Psychiatry.

[21] Alvarez-Jimenez M, Parker AG, Hetrick SE, McGorry PD, Gleeson JF (2011). Preventing the second episode: a systematic review and meta-analysis of psychosocial and pharmacological trials in first-episode psychosis. Schizophrenia Bull..

[22] Pina-Camacho L (2016). Age at first episode modulates diagnosis-related structural brain abnormalities in psychosis. Schizophrenia Bull..

[23] Berge, D. et al. Elevated extracellular free-water in a multicentric first-episode psychosis sample, decrease during the first 2 years of illness. *Schizophrenia Bull.*10.1093/schbul/sbz132 (2020). 10.1093/schbul/sbz132 PMC7342177 31915835

[24] Radua J, Grunze H, Amann BL (2017). Meta-analysis of the risk of subsequent mood episodes in bipolar disorder. Psychother. Psychosomatics.

[25] Andreasen NC (2005). Remission in schizophrenia: proposed criteria and rationale for consensus. Am J Psychiatry.

[26] Salvador R (2017). Evaluation of machine learning algorithms and structural features for optimal MRI-based diagnostic prediction in psychosis. PLoS ONE.

[27] Fortin JP (2018). Harmonization of cortical thickness measurements across scanners and sites. NeuroImage.

[28] Radua J (2020). Increased power by harmonizing structural MRI site differences with the ComBat batch adjustment method in ENIGMA. NeuroImage.

[29] Solanes A (2021). Biased accuracy in multisite machine-learning studies due to incomplete removal of the effects of the site. Psychiatry Res. Neuroimaging.

[30] Radua J (2014). Anisotropic kernels for coordinate-based meta-analyses of neuroimaging studies. Front. Psychiatry.

[31] Landin-Romero R (2016). Midline brain abnormalities across psychotic and mood disorders. Schizophrenia Bull..

[32] Kasai K (2004). Cavum septi pellucidi in first-episode schizophrenia and first-episode affective psychosis: an MRI study. Schizophrenia Res..

[33] Greenshtein E, Ritov YA (2004). Persistence in high-dimensional linear predictor selection and the virtue of overparametrization. Bernoulli.

[34] Radua J (2021). What is the actual accuracy of clinical prediction models? The case of transition to psychosis. Neurosci. Biobehavioral Rev..

[35] Young J, Kempton MJ, McGuire P (2016). Using machine learning to predict outcomes in psychosis. Lancet Psychiatry.

[36] Oliver D, Radua J, Reichenberg A, Uher R, Fusar-Poli P (2019). Psychosis polyrisk score (PPS) for the detection of individuals at-risk and the prediction of their outcomes. Front. Psychiatry.

[37] Radua J (2018). What causes psychosis? An umbrella review of risk and protective factors. World Psychiatry.

[38] Oliver D (2020). Real-world digital implementation of the psychosis polyrisk score (PPS): a pilot feasibility study. Schizophrenia Res..

[39] Mondelli V (2020). Baseline high levels of complement component 4 predict worse clinical outcome at 1-year follow-up in first-episode psychosis. Brain Behav. Immunity.

[40] Harrisberger F (2016). Impact of polygenic schizophrenia-related risk and hippocampal volumes on the onset of psychosis. Transl. Psychiatry.

[41] Baecker L (2021). Brain age prediction: a comparison between machine learning models using region- and voxel-based morphometric data. Human Brain Mapping.

[42] Simhandl C, Radua J, Konig B, Amann BL (2015). The prevalence and effect of life events in 222 bipolar I and II patients: a prospective, naturalistic 4 year follow-up study. J. Affective Disorders.

[43] Robinson D (1999). Predictors of relapse following response from a first episode of schizophrenia or schizoaffective disorder. Archives General Psychiatry.

[44] Tiihonen J (2011). A nationwide cohort study of oral and depot antipsychotics after first hospitalization for schizophrenia. Am. J. Psychiatry.

[45] Nieuwenhuis M (2012). Classification of schizophrenia patients and healthy controls from structural MRI scans in two large independent samples. NeuroImage.

