# Supplementary material for: Combining MRI and clinical data to detect high relapse risk after the first episode of psychosis
Source: Schizophrenia (Heidelb). 2022 Nov 17;8(1):100. doi: 10.1038/s41537-022-00309-w (PMC9672064; doi:10.1038/s41537-022-00309-w)
Supplement: Supplementary file 1 — Supplement material [file 41537_2022_309_MOESM1_ESM.docx]

**Combining MRI and clinical data to detect**

**High Relapse Risk after the First Episode of Psychosis**

**Supplement**

**
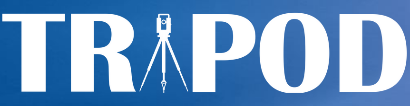
TRIPOD Checklist: Prediction Model Development and Validation**

| **Section/Topic** | **Item** |  | **Checklist Item** | **Page** |
| --- | --- | --- | --- | --- |
| **Title and abstract** | | | | |
| Title | 1 | D;V | Identify the study as developing and/or validating a multivariable prediction model, the target population, and the outcome to be predicted. | 1 |
| Abstract | 2 | D;V | Provide a summary of objectives, study design, setting, participants, sample size, predictors, outcome, statistical analysis, results, and conclusions. | 3 |
| **Introduction** | | | | |
| Background and objectives | 3a | D;V | Explain the medical context (including whether diagnostic or prognostic) and rationale for developing or validating the multivariable prediction model, including references to existing models. | 4 |
|  | 3b | D;V | Specify the objectives, including whether the study describes the development or validation of the model or both. | 4 |
| **Methods** | | | | |
| Source of data | 4a | D;V | Describe the study design or source of data (e.g., randomized trial, cohort, or registry data), separately for the development and validation data sets, if applicable. | 5-6 |
|  | 4b | D;V | Specify the key study dates, including start of accrual; end of accrual; and, if applicable, end of follow-up. | Suppl. 5 |
| Participants | 5a | D;V | Specify key elements of the study setting (e.g., primary care, secondary care, general population) including number and location of centres. | 5, Suppl. 5-6 |
|  | 5b | D;V | Describe eligibility criteria for participants. | Suppl. 5 |
|  | 5c | D;V | Give details of treatments received, if relevant. | No treatment received |
| Outcome | 6a | D;V | Clearly define the outcome that is predicted by the prediction model, including how and when assessed. | 6-7, Suppl. 5 |
|  | 6b | D;V | Report any actions to blind assessment of the outcome to be predicted. | 6-7, Suppl. 5 |
| Predictors | 7a | D;V | Clearly define all predictors used in developing or validating the multivariable prediction model, including how and when they were measured. | 6-7, Suppl. 5 |
|  | 7b | D;V | Report any actions to blind assessment of predictors for the outcome and other predictors. | 6-7, Suppl. 5 |
| Sample size | 8 | D;V | Explain how the study size was arrived at. | 5, Suppl. 5-6 |
| Missing data | 9 | D;V | Describe how missing data were handled (e.g., complete-case analysis, single imputation, multiple imputation) with details of any imputation method. | Suppl. 5 |
| Statistical analysis methods | 10a | D | Describe how predictors were handled in the analyses. | 5-6 |
|  | 10b | D | Specify type of model, all model-building procedures (including any predictor selection), and method for internal validation. | 5-7 |
|  | 10c | V | For validation, describe how the predictions were calculated. | 5-8 |
|  | 10d | D;V | Specify all measures used to assess model performance and, if relevant, to compare multiple models. | 6-7 |
|  | 10e | V | Describe any model updating (e.g., recalibration) arising from the validation, if done. | Not done |
| Risk groups | 11 | D;V | Provide details on how risk groups were created, if done. | 7 |
| Development vs. validation | 12 | V | For validation, identify any differences from the development data in setting, eligibility criteria, outcome, and predictors. | Same data (cross-validation) |
| **Results** | | | | |
| Participants | 13a | D;V | Describe the flow of participants through the study, including the number of participants with and without the outcome and, if applicable, a summary of the follow-up time. A diagram may be helpful. | 7, Suppl. 5 |
|  | 13b | D;V | Describe the characteristics of the participants (basic demographics, clinical features, available predictors), including the number of participants with missing data for predictors and outcome. | 5,7, Suppl. 5 |
|  | 13c | V | For validation, show a comparison with the development data of the distribution of important variables (demographics, predictors, and outcome). | Same data (cross-validation) |
| Model development | 14a | D | Specify the number of participants and outcome events in each analysis. | 8 |
|  | 14b | D | If done, report the unadjusted association between each candidate predictor and outcome. | Supplement |
| Model specification | 15a | D | Present the full prediction model to allow predictions for individuals (i.e., all regression coefficients, and model intercept or baseline survival at a given time point). | Supplement and website |
|  | 15b | D | Explain how to the use the prediction model. | Supplement and website |
| Model performance | 16 | D;V | Report performance measures (with CIs) for the prediction model. | 8-9 |
| Model-updating | 17 | V | If done, report the results from any model updating (i.e., model specification, model performance). | Not done |
| **Discussion** | | | | |
| Limitations | 18 | D;V | Discuss any limitations of the study (such as nonrepresentative sample, few events per predictor, missing data). | 10-11 |
| Interpretation | 19a | V | For validation, discuss the results with reference to performance in the development data, and any other validation data. | Same data (cross-validation) |
|  | 19b | D;V | Give an overall interpretation of the results, considering objectives, limitations, results from similar studies, and other relevant evidence. | 9-10 |
| Implications | 20 | D;V | Discuss the potential clinical use of the model and implications for future research. | 9-10 |
| **Other information** | | | | |
| Supplementary information | 21 | D;V | Provide information about the availability of supplementary resources, such as study protocol, Web calculator, and data sets. | 5-9 |
| Funding | 22 | D;V | Give the source of funding and the role of the funders for the present study. | 12 |

**Description of the cohort and follow-up**

The cohort included 227 patients with a FEP. Of them, 186 were from the multicenter PEPs-Img study ([1-3](#_ENREF_1)) recruited between April 2009 and April 2011. We recruited the additional 41 patients to achieve a larger cohort size between Feb 2017 and Mar 2019 from Benito Menni CASM, Hospital General de Granollers for this study, Hospital de Mataró, and Hospital Sant Rafael. The recruitment of new participants consisted of nearly identical inclusion criteria to make them comparable to those previously recruited. The PEPs-Img study recruited 196 individuals with a FEP scanned in 6 devices between January 2009 and December 2011. The recruitment included patients with a FEP of any kind. Patients were prospectively followed for two years, but we had to discard ten individuals because we could not determine their time to relapse, resulting in a final sample size of 186 subjects from the PEPs-Img study and 41 newly recruited.

Exclusion criteria for all patients were: a) history of head trauma with loss of consciousness; b) intellectual disability; c) systemic disease with mental health impact; and d) contraindications to MRI (e.g., metallic implants, claustrophobia, pregnancy). Two additional exclusion criteria for the PEPs-Img individuals were: a) age <12 or >35 years; and b) duration of illness >12 months. Three other exclusion criteria for the newly recruited individuals were: a) age <18 or >65 years; b) duration of illness >18 months; c) non-righthandedness; and d) substance dependence during the previous year.

Experienced psychiatrists, trained in the assessment tools, collected the clinical data. They established the DSM-IV diagnoses using the Structured Clinical Interview for DSM-IV Axis I Disorders (SCID-I) ([4](#_ENREF_4)), or the Kiddie-SADS Present and Lifetime Version (K-SADS-PL) ([5](#_ENREF_5)) for individuals under 18 years, and gathered information from medical records and conducted interviews with relevant informants where appropriate. In addition to the Positive and Negative Syndrome Scale (PANSS) ([6](#_ENREF_6)), they measured the severity with the Clinical Global Impression-Severity scale (CGI-S) ([7](#_ENREF_7)), the baseline level of functioning with the Global Assessment of Functioning scale (GAF) ([8](#_ENREF_8)), or the Children's Global Assessment Scale (CGAS) ([9](#_ENREF_9)) for individuals under 18 years, and affective symptoms with the Young Mania Rating Scale (YMRS) ([10](#_ENREF_10)) and the Montgomery-Asberg Depression Rating Scale (MADRS) ([11](#_ENREF_11)). Finally, we imputed the missing clinical data 20 times using "lasso" (least absolute shrinkage and selection operator) regressions ([12](#_ENREF_12)).

The mean age of the 227 patients with a FEP was 24.2 years (SD 7.4), and there were 78 females (34.4%). Sex was determined by the researcher at the clinical interview without disagreement with the patient. The average years of education were 10.4 (SD 3.2). A little more than half (58.2%) had a family psychiatric history, including 28.6% affective history and 1.4% suicide. There were 48 affective psychosis (21.1%), and the diagnosis at baseline were schizophrenia (n = 78, 34.4%), bipolar disorder (n = 41, 18.1%), schizoaffective disorder (n = 12, 5.3%), substance-induced psychosis (n = 10, 4.4%), major depressive disorder (n = 5, 2.2%), and others (brief psychotic disorder, schizophreniform disorder, delusional disorder, and psychotic disorder not otherwise specified; n = 81, 35.7%). The average PANSS score was 71.2 (SD 24.4), with 17.6 (SD 7.9) for the positive scale, 18.2 (SD 8.5) for the negative scale, and 35.9 (SD 12.7) for the general psychopathology scale. Patients' functioning was moderate to seriously impaired (GAF 50.5, SD 19.7). They were moderate to markedly ill (CGI-S 4.4, SD 1.1). The average YMRS score was 7.9 (SD 10.2), and the average MADRS score was 12.2 (SD 9.9).

We defined relapses as exacerbations of symptoms during at least one week with at least one of eight PANSS items (P1, P2, P3, N1, N4, N6, G5, and G9) scoring above 3 (mild) ([13](#_ENREF_13)). For PEPs-Img patients, the visits were at 2, 6, 12, and 24 months after the MRI; if there was a relapse between two visits, we used the date in the middle of the interval between the two visits. The follow-up visits were at 2, 4, 6, 9, 12, 15, and 18 months after the MRI for newly recruited patients; if the relapse occurred between two visits, the psychiatrist wrote the approximate relapse date. For all patients with no relapse during the follow-up, we saved the date of the last visit. Then, finally, we measured the time from the scan to the relapse or last visit.

On the contrary, remission was defined as scoring <3 in all eight PANSS items (P1, P2, P3, N1, N4, N6, G5, and G9). We only considered relapse after at least six months of remission.

The ethical committees of all hospitals involved (Benito Menni CASM, Hospital General de Granollers, Hospital de Mataró, Hospital Sant Rafael, Hospital de Bellvitge, Hospital Clínic i Provincial de Barcelona, Hospital Universitario 12 de Octubre, Hospital Clínic de València, Hospital del Mar, Instituto de Investigación Sanitaria Aragón, Hospital General Universitario Gregorio Marañón, Hospital Sant Joan de Déu Barcelona, Hospital Santiago Apóstol de Vitoria-Gasteiz) had approved the study, conducted according to the Declaration of Helsinki.

**MRI devices and sequences**

| Site | Cohort size | Device | Sequence |
| --- | --- | --- | --- |
| PEPs-Img Barcelona | n=99 | 3T Siemens Trio TIM | TR=2300ms; TE=2.98ms; flip angle=9º; 240 sagittal slices; slice thickness=1mm; matrix size=256×256; 1×1×1mm^3^ voxel resolution |
| PEPs-Img Madrid | n=26 | 1.5T Philips Intera | TR=25ms; TE=9.18ms; flip angle=30º; 175 sagittal slices; slice thickness=1mm; matrix size=240×240; 1×0.94×0.94mm^3^ voxel resolution |
| PEPs-Img Zaragoza | n=22 | 1.5T GE Signa Excite | TR=9.28ms; TE=1.9ms; flip angle=20º; 156 sagittal slices; slice thickness=1mm; matrix size=256×256; 1×0.94×0.94mm^3^ voxel resolution |
| PEPs-Img Vitoria | n=21 | 1.5T Siemens Avanto | TR=25ms; TE=9.21ms; flip angle=30º; 176 sagittal slices; slice thickness=1mm; matrix size=256×256; 1×0.95×0.95mm^3^ voxel resolution |
| PEPs-Img Asturias | n=13 | 1.5T GE Genesis Signa | TR=31ms; TE=2.9ms; flip angle=30º; 152 sagittal slices; slice thickness=1mm; matrix size=256×256; 1×1×1mm^3^ voxel resolution |
| PEPs-Img Valencia | n=5 | 3T Philips Achieva | TR=8.37ms; TE=3.87ms; flip angle=8º; 160 transversal slices; slice thickness=1mm; matrix size=256×256; 0.94×0.94×1mm^3^ voxel resolution |
| Newly recruited participants | n=41 | 3T Philips Ignenia | TR=9.9ms; TE=4.6ms; flip angle=8º; 180 axial slices; slice thickness=1mm; matrix size=240×240; 1×1×1mm^3^ voxel resolution |

**Processing of baseline structural MRI data**

We visually inspected the images to detect abnormalities or artifacts, segmented them into gray matter, white matter, and several other tissue classes ([14](#_ENREF_14)), non-linearly registered the segments to the MNI space with SPM12 (Wellcome Trust Center for Neuroimaging, London, UK). However, the non-linear registration did not include creating a study-specific template or any approach requiring input from the whole cohort. The reason is that we wanted to simulate the scenario in which the clinicians would use the tool to detect future patients at HRR-FEP. In this scenario, the tool creators could not create a template using the future patients' images. Finally, according to the results of the optimization of parameters (see later), we saved both unmodulated and modulated images, applied standard smoothing kernel (σ=4mm, corresponding to FWHM≈10.5mm), and subsampled the data three times with FSL 5, obtaining 12×12×12 mm^3^ voxels.

**Datasets used for optimizing the MRI-based machine learning parameters**

*Age predictions:* We included 120 healthy individuals (50% females, mean±SD age=48.7±16.5), which we had previously selected from the IXI dataset (<http://www.brain-development.org/>) to form an age- and sex-balanced sample: 20 males and 20 females between 20 and 40 years, between 40 and 60 years, and between 60 and 80 years.

Researchers at Hammersmith Hospital had acquired a high-resolution structural image with a 3T Philips device (Philips Medical Systems, Best, the Netherlands) from each participant. They used a T1-weighted sequence (TR=9.6ms; TE=4.6ms; flip angle=8º: 150 sagittal slices; slice thickness=1mm with no gap; matrix size=256×256; 1×1×1mm^3^ voxel resolution).

*Diagnostic predictions:* The included 128 patients and the 127 controls were matched for age (mean±SD=41.5±10.3 and 39.8±10.3), sex (42% and 43% females), and estimated premorbid intelligence quotient (measured as TAP([15](#_ENREF_15))±SD: 22.1±4.8 and 23.0±4.4). We had recruited the patients for previous studies at FIDMAG Research Unit, Barcelona. The healthy controls were non-medical hospital staff, relatives and acquaintances, and independent sources in the community. All individuals were right-handed, 18-65 years old, had no history of brain trauma or neurological disease, and had no substance abuse in the last 12 months. In addition, healthy controls had no history of mental disorders or treatment with psychotropic medication. All participants had given written informed consent, and the Ethics Committee of the hospitals had approved the studies. Experienced psychiatrists had established the schizophrenia diagnosis with the Structured Clinical Interview for DSM-IV Axis I Disorders (SCID-I) ([4](#_ENREF_4)). They had also measured the severity of its symptoms with the Positive and Negative Syndrome Scale (PANSS) ([6](#_ENREF_6)) (total score±SD=72.6±17.5; positive score±SD=16.9±5.7; negative score±SD=21.4±7.0; general psychopathology score±SD=34.3±8.4). We refer the reader to the previous studies for further details ([16-19](#_ENREF_16)).

We acquired a high-resolution structural image from each participant with a 1.5T GE Signa device (General Electric Medical Systems, Milwaukee, WI, USA). We used a T1-weighted sequence (TR=2000ms; TE=4ms; flip angle=15º; 180 axial slices; slice thickness=1mm with no gap; matrix size=512×512; 0.5×0.5×1mm^3^ voxel resolution).

*Analyses:* The analyses were analogous to those described in the main paper for the first episode of psychosis (FEP) cohort. However, there was no need to account for the effects of the site because the datasets were single sited; we did not remove the effects of age from MRI data in the dataset used for age predictions; the outcome to predict was the age or the diagnosis; and the independent variables were only the MRI data.

**Univariate analysis of the brain regions and clinical variables associated with relapse**

First, we removed the effects of the site, age, and sex from the segmented image (e.g., the modulated gray matter). To this end, we applied ComBat to remove the effects of the site, fitted a linear model for each voxel for age and sex, and saved the residual. The dependent variable was the voxel's value (e.g., the gray matter volume), and the independent variables were age and sex.

Second, we fitted a Cox proportional hazards regression model, again separately for each voxel. The dependent variable was the time to relapse (or to the last visit for patients with no relapse recorded). The independent variables were the voxels' values. We saved the z-value and p-value corresponding to the effects of the residual value of the voxel. We did not keep the hazard ratios (HR) to avoid confusion. In voxel-based analyses, peaks are an artificially selected population of voxels with exceptionally high values. We report peak z-values because we assume that the reader has already seen high peak z-values in other papers. However, we think that reporting high peak HR (e.g., >100) could erroneously give the impression of very relevant effects – while they may be somewhat irrelevant.

Finally, we applied a relatively liberal statistical threshold (uncorrected p<0.005) to the results. We only aimed to show descriptive maps of brain region regions potentially associated with increased or decreased risk of relapse and considered this threshold adequate. We did not specify a minimum cluster extent because voxels were already very large (one voxel = 1.7cm^3^).

We produced four maps: unmodulated gray matter, modulated gray matter, unmodulated white matter, and modulated white matter. We conducted these analyses with an R script that we provide somewhere else ([20](#_ENREF_20)) and the "imgcalc" tool ([21](#_ENREF_21)).

To detect clinical variables potentially associated with relapse, we conducted simple Cox proportional hazards regressions. The dependent variable was the time to relapse (or to the last visit for patients with no relapse recorded). The independent variables were the clinical information. We report those clinical variables with uncorrected p<0.05. Again, we did not correct for multiple comparisons because this analysis's aim was exclusively descriptive. We repeated the cross-validation ten times, each time with a different cohort division in folds.

**The entire machine learning model**

In the following table, we report the models obtained from the 10-fold cross validation and the whole sample. These two models are both available to use in the web application provided to detect HRR-FEP.

|  | | 10-fold model | Whole sample model |
| --- | --- | --- | --- |
| Clinical variables | |  |  |
|  | Schizoaffective disorder | β=+0.24 | β=+0.08 |
|  | ↓ Difficulty in abstract thinking (PANSS N5) | β=-0.07 | β=-0.07 |
|  | ↓ PANSS Negative total | β=-0.002 | β=-0.003 |
|  | ↓ Uncooperativeness (PANSS G8) | β=-0.01 | β=-0.003 |
|  | ↓ Poor attention (PANSS G11) | β=-0.04 | β=-0.009 |
|  | ↓ Poor impulse control (PANSS G14) | β=-0.01 | β=-0.002 |
|  | ↑ Pessimistic thoughts (MADRS 9) | β=+0.0044 | β=+0.04 |
|  | ↑ Speech (YMRS 6) | β=+0.002 | β=+0.002 |
|  | ↑ Excitement (PANNS P4) | β=+0.0015 | - |
|  | ↑ Anxiety (PANNS G2) | β=+0.0011 | - |
|  | ↑ Lassitude (MADRS 7) | β=+0.001 | - |
|  | ↓ MADRS Total | β=-0.0007 | - |
|  | ↑ Reduced appetite (MADRS 5) | β=+0.0006 | - |
|  | ↓ Hostility (PANNS P7) | β=-0.0005 | - |
|  | ↓ Global Assessment of Functioning (GAF) | β=-0.0003 | - |
|  | ↑ Elevated Mood (YMSR 1) | β=+0.0003 | - |
|  | ↑ A.NAF not affective | β=+0.0001 | - |
|  | ↓ Disorientation (PANNS G10) | β=-0.0001 | - |
|  | ↓ TDM diagnosis | β=-0.0001 | - |
|  | ↓ Grandiosity (PANNS P5) | β=-0.0043 | - |
|  | ↑ Appearance (YMRS 10) | β=+0.0028 | - |
|  | ↑ Somatic concern (PANNS G1) | β=+0.0027 | - |
|  | ↑ Sexual Interest (YMRS 3) | β=+0.0027 | - |
|  | ↓ Stereotyped thinking (PANNS N7) | β=-0.0048 | - |
|  | ↓ Conceptual Disorganization (PANNS P2) | β=-0.01 | - |
|  | ↓ Poor rapport (PANNS N3) | β=-0.01 | - |
|  | ↑ Injectable Antipsychotic | β=+0.01 | - |
| Gray matter increase | |  |  |
|  | ↑ R Lingual | Unm, [6, -78, -12], β=+0.05 | Unm, [6, -78, -12], β=+0.04 |
|  | ↑ R Postcentral | Unm, [54, -6, 24], β=+0.1 | Unm, [54, -6, 24], β=+0.03 |
|  | ↑ Cerebelum_7b_R | Unm, [6,-78,-48], β=+0.005 | Unm, [6, -78, -48], β=+0.001 |
|  | ↑ L Putamen | Unm, [-18,18,0], β=+0.0048 | - |
|  | ↑ L Amygdala | Unm, [-30,-6,-12], β=+0.0037 | - |
|  | ↑ L Cerebelum | Unm, [-30,-66,-60], β=+0.0032 | - |
|  | ↑ L Olfactory | Unm, [-6,30,0], β=+0.0028 | - |
|  | ↑ R Supplementary Motor Area | Unm, [6,-6,72], β=+0.0014 | - |
|  | ↑ L Cingulum Anterior | Unm, [-6,42,12], β=+0.0011 | - |
|  | ↑ R Cerebelum | Unm, [6,-90,-24], β=+0.0009 | - |
|  | ↑ R Putamen | Unm, [18,18,0], β=+0.0005 | - |
|  | ↑ R Olfactory | Mod, [6,30,0], β=+0.0004 | - |
|  | ↑ R Cerebelum | Unm, [6,-66,-48], β=+0.0004 | - |
|  | ↑ R Cerebelum | Unm, [30,-66,-60], β=+0.0003 | - |
|  | ↑ R Cerebelum | Unm, [30,-54,-60], β=+0.0003 | - |
|  | ↑ R Olfactory | Unm, [6,30,0], β=+0.0002 | - |
|  | ↑ R Frontal | Unm, [30,30,-24], β=+0.0001 | - |
|  | ↑ R Cuneus | Unm, [18,-66,36], β=+0.0001 | - |
|  | ↑ R Cerebelum | Unm, [6,-78,-36], β=<0.0001 | - |
| Gray matter decrease | |  |  |
|  | ↓ R Middle temporal | Unm, [66, -6, -12], β=-0.43 | Unm, [66, -6, -12], β=-0.34 |
|  | ↓ R Inferior frontal | Unm, [30, 6, 36], β=-0.21 | Unm, [30, 6, 36], β=-0.12 |
|  | ↓ R Middle frontal | Unm, [30, 42, 36], β=-0.20 | Unm, [30, 42, 36], β=-0.23 |
|  | ↓ R Rectus | Unm, [6, 30, -24], β=-0.15 | Unm, [6, 30, -24], β=-0.11 |
|  | ↓ R Rectus | Mod, [6, 30, -24], β=-0.17 | Mod, [6, 30, -24], β=-0.20 |
|  | ↓ R Precentral | Mod, [42, 6, 36], β=-0.18 | Mod, [42, 6, 36], β=-0.17 |
|  | ↓ R Angular | Unm, [30, -54, 36], β=-0.06 | Unm, [30,-54,36], β=-0.02 |
|  | ↓ L Precentral | Mod, [-42,6,36] , β=-0.01 | Mod, [-42,6,36], β=-0.0002 |
|  | ↓ L Middle Frontal | Unm, [-30,6,36] , β=-0.0037 | - |
|  | ↓ R Insula | Unm, [30,30,0] , β=-0.0021 | - |
|  | ↓ L Cerebelum | Mod, [-18,-90,-36] , β=-0.0015 | - |
|  | ↓ R Middle Frontal | Unm, [18,66,-12] , β=-0.0012 | - |
|  | ↓ R Inferior Frontal | Unm, [54,18,36] , β=-0.0008 | - |
|  | ↓ R Middle Temporal | Mod, [66,-6,-12] , β=-0.0006 | - |
|  | ↓ R Middle Frontal | Unm, [42,6,60] , β=-0.0004 | - |
|  | ↓ L Inferior Parietal | Unm, [-30,-42,36] , β=-0.0004 | - |
|  | ↓ R Middle Temporal | Mod, [66,-42,12] , β=-0.0003 | - |
|  | ↓ L Calcarine | Unm, [-6,-102,-12] , β=-0.0003 | - |
|  | ↓ L Inferior Parietal | Unm, [-30,-30,36] , β=-0.0002 | - |
|  | ↓ L Postcentral | Unm, [-42,-42,60] , β=-0.0002 | - |
|  | ↓ R Precentral | Unm, [54,6,36] , β=-0.0002 | - |
|  | ↓ R Superior Frontal | Unm, [18,6,72] , β=-0.0002 | - |
|  | ↓ R Middle Frontal | Unm, [30,66,-12] , β=-0.0002 | - |
|  | ↓ L Postcentral | Unm, [-30,-30,72] , β=-0.0001 | - |
|  | ↓ R Middle Frontal | Mod, [42,6,60] , β=-0.0001 | - |
|  | ↓ L Precentral | Unm, [-42,6,36] , β=-0.0001 | - |
|  | ↓ L Inferior Frontal | Mod, [-42,30,24] , β=-0.0001 | - |
|  | ↓ R Cerebelum | Unm, [30,-54,-48] , β=-0.0001 | - |
|  | ↓ L Cerebelum | Unm, [-6,-54,-36] , β=-0.0001 | - |
| White matter increase | |  |  |
|  | ↑ R Precentral | Unm, [42,6,36], β=+0.54 | Unm, [42,6,36], β=+0.58 |
|  | ↑ L Middle frontal | Unm, [-42, 6, 36], β=+0.10 | Unm, [-42, 6, 36], β=+0.03 |
|  | ↑ R Angular | Unm, [30,-54,36], β=+0.03 | Unm, [30,-54,36], β=+0.02 |
|  | ↑ R Inferior Frontal | Unm, [30,6,36], β=+0.03 | Unm, [30,6,36], β=+  0.006 |
|  | ↑ R Inferior Parietal | Unm, [30,-54,48], β=+0.02 | Unm, [30,-54,48], β=+0.006 |
|  | ↑ L Inferior Frontal | Unm, [-42,30,24], β=+0.0035 | Unm, [-42,30,24], β=+0.001 |
|  | ↑ R Insula | Unm, [30,30,0], β=+0.02 | - |
|  | ↑ L Inferior Frontal | Unm, [-42,18,36], β=+0.004 | - |
|  | ↑ L Inferior Parietal | Unm, [-42,-42,36], β=+0.0037 | - |
|  | ↑ L Middle Frontal | Unm, [-30,6,36], β=+0.0029 | - |
|  | ↑ L Fusiform | Unm, [-30,-78,-12], β=+0.0023 | - |
|  | ↑ L Middle Frontal | Unm, [-30,18,48], β=+0.0021 | - |
|  | ↑ R Superior Frontal | Unm, [18,30,48], β=+0.0019 | - |
|  | ↑ L Precentral | Unm, [-42,-6,36], β=+0.0014 | - |
|  | ↑ L Inferior Frontal | Unm, [-42,30,0], β=+0.0011 | - |
|  | ↑ L Superior Parietal | Unm, [-30,-66,48], β=+0.001 | - |
|  | ↑ R Inferior Frontal | Unm, [42,18,36], β=+0.0008 | - |
|  | ↑ L Superior Frontal | Unm, [-18,-6,60], β=+0.0007 | - |
|  | ↑ R Hippocampus | Unm, [30,-42,12], β=+0.0003 | - |
|  | ↑ R Inferior Temporal | Unm, [54,-18,-24], β=+0.0002 | - |
|  | ↑ L Inferior Parietal | Unm, [-30,-42,36], β=+0.0001 | - |
|  | ↑ L Precentral | Mod, [-18,-18,72], β=+0.0001 | - |
| White matter decrease | |  |  |
|  | ↓ L Inferior frontal | Mod, [-42, 18, 12], β=-0.73 | Mod, [-42, 18, 12], β=-1.08 |
|  | ↓ L Inferior frontal | Unm, [-42, 18, 12], β=-0.57 | Unm, [-42, 18, 12], β=-0.54 |
|  | ↓ R Middle frontal | Unm, [30, 30, 36], β=-0.86 | Unm, [30,30,36], β=-0.78 |
|  | ↓ R Cuneus | Mod, [18, -90, 12], β=-0.18 | Mod, [18, -90, 12], β=-0.08 |
|  | ↓ R Postcentral | Mod, [54, -6, 24], β=-0.09 | Mod, [54, -6, 24], β=-0.06 |
|  | ↓ L corpus callosum | Unm, [-18, -30, 24], β=-0.07 | Unm, [-18,-30,24], β=-0.02 |
|  | ↓ L Middle frontal | Mod, [-30, 42, 12], β=-0.07 | - |
|  | ↓ R corpus callosum | Mod, [6, 30, 0], β=-0.06 | Mod, [6, 30, 0], β=-0.04 |
|  | ↓ R Middle CIngulum | Mod, [18,-18,48] wm, β=-0.03 | Mod, [18,-18,48], β=-0.02 |
|  | ↓ L Angular | Unm, [-42,-54,36] wm, β=-0.02 | Unm, [-42,-54,36], β=-0.01 |
|  | ↓ R Postcentral | Unm, [54,-6,24] wm, β=-0.01 | Unm, [54,-6,24], β=-0.005 |
|  | ↓ L Thalamus | Unm, [-6,-18,24] wm, β=-0.01 | Unm, [-6,-18,24], β=-0.0004 |
|  | ↓ L Middle Frontal | Mod, [-30,42,12] wm, β=-0.06 | - |
|  | ↓ R Olfactory | Mod, [6,30,0] wm, β=-0.05 | - |
|  | ↓ L Caudate | Unm, [-18,-30,24] wm, β=-0.05 | - |
|  | ↓ R Cuneus | Unm, [18,-66,36] wm, β=-0.04 | - |
|  | ↓ L Superior Parietal | Unm, [-30,-54,60] wm, β=-0.02 | - |
|  | ↓ L Postcentral | Mod, [-54,-6,24] wm, β=-0.02 | - |
|  | ↓ L Angular | Mod, [-42,-54,36] wm, β=-0.01 | - |
|  | ↓ L Lingual | Mod, [-18,-90,0] wm, β=-0.01 | - |
|  | ↓ L Superior Occipital | Mod, [-18,-66,24] wm, β=-0.01 | - |
|  | ↓ R Middle Frontal | Mod, [30,42,12] wm, β=-0.01 | - |
|  | ↓ R Cuneus | Mod, [18,-66,36] wm, β=-0.01 | - |
|  | ↓ L Caudate | Mod, [-18,-30,24] wm, β=-0.01 | - |
|  | ↓ R Middle Temporal | Mod, [42,-54,0] wm, β=-0.0047 | - |
|  | ↓ R Superior Frontal | Mod, [18,42,0] wm, β=-0.0045 | - |
|  | ↓ R Inferior Frontal | Unm, [42,18,24] wm, β=-0.0041 | - |
|  | ↓ R Inferior Frontal | Mod, [42,18,24] wm, β=-0.004 | - |
|  | ↓ R Calcarine | Mod, [18,-78,12] wm, β=-0.0035 | - |
|  | ↓ R Calcarine | Unm, [18,-102,0] wm, β=-0.0026 | - |
|  | ↓ L Calcarine | Mod, [-18,-78,12] wm, β=-0.0025 | - |
|  | ↓ R Thalamus | Unm, [6,-6,24] wm, β=-0.0025 | - |
|  | ↓ L Thalamus | Unm, [-6,-6,24] wm, β=-0.0022 | - |
|  | ↓ R Calcarine | Mod, [18,-90,0] wm, β=-0.0018 | - |
|  | ↓ L Amygdala | Unm, [-30,-6,-12] wm, β=-0.0018 | - |
|  | ↓ L Cingulum Anterior | Mod, [-18,42,0] wm, β=-0.0017 | - |
|  | ↓ L Superior Occipital | Unm, [-18,-66,24] wm, β=-0.0016 | - |
|  | ↓ R Angular | Mod, [42,-42,24] wm, β=-0.0013 | - |
|  | ↓ R Middle Temporal | Mod, [42,-54,12] wm, β=-0.0013 | - |
|  | ↓ L Postcentral | Unm, [-54,-6,24] wm, β=-0.0013 | - |
|  | ↓ R Caudate | Unm, [18,-6,24] wm, β=-0.0012 | - |
|  | ↓ R Angular | Mod, [30,-42,36] wm, β=-0.0008 | - |
|  | ↓ R Thalamus | Unm, [6,-18,24] wm, β=-0.0007 | - |
|  | ↓ R Caudate | Unm, [18,-30,24] wm, β=-0.0007 | - |
|  | ↓ R Superior Parietal | Unm, [30,-42,48] wm, β=-0.0005 | - |
|  | ↓ L Lingual | Unm, [-18,-90,0] wm, β=-0.0003 | - |
|  | ↓ R Superior Frontal | Mod, [18,30,-12] wm, β=-0.0002 | - |
|  | ↓ L Middle Occipital | Unm, [-18,-102,0] wm, β=-0.0002 | - |
|  | ↓ L Postcentral | Unm, [-30,-30,60] wm, β=-0.0002 | - |
|  | ↓ L Superior Temporal | Unm, [-54,-42,24] wm, β=-0.0002 | - |
|  | ↓ R SupraMarginal | Mod, [42,-30,36] wm, β=-0.0001 | - |
|  | ↓ R Precentral | Unm, [54,6,24] wm, β=-0.0001 | - |
|  | ↓ R Caudate | Mod, [18,-30,24] wm, β=-0.0001 | - |
|  | ↓ R Middle Temporal | Unm, [42,-54,12] wm, β=-0.0001 | - |
|  | ↓ R Middle Frontal | Mod, [30,30,36] wm, β=-0.0001 | - |

**References**

1. Bernardo M, Bioque M, Parellada M, Saiz Ruiz J, Cuesta MJ, Llerena A, et al. Assessing clinical and functional outcomes in a gene-environment interaction study in first episode of psychosis (PEPs). Rev Psiquiatr Salud Ment. 2013;6(1):4-16.

2. Pina-Camacho L, Del Rey-Mejias A, Janssen J, Bioque M, Gonzalez-Pinto A, Arango C, et al. Age at First Episode Modulates Diagnosis-Related Structural Brain Abnormalities in Psychosis. Schizophrenia bulletin. 2016;42(2):344-57.

3. Berge D, Mane A, Lesh TA, Bioque M, Barcones F, Gonzalez-Pinto AM, et al. Elevated Extracellular Free-Water in a Multicentric First-Episode Psychosis Sample, Decrease During the First 2 Years of Illness. Schizophrenia bulletin. 2020.

4. First M, Spitzer RL, Gibbon M, Williams JBW. Structured Clinical Interview for DSM-IV-TR Axis I Disorders, Research Version, Patient Edition. (SCID-I/P). New York, NY: Biometrics Research, New York State Psychiatric Institute; 2002.

5. Kaufman J, Birmaher B, Brent D, Rao U, Flynn C, Moreci P, et al. Schedule for Affective Disorders and Schizophrenia for School-Age Children-Present and Lifetime Version (K-SADS-PL): initial reliability and validity data. J Am Acad Child Adolesc Psychiatry. 1997;36(7):980-8.

6. Kay SR, Fiszbein A, Opler LA. The positive and negative syndrome scale (PANSS) for schizophrenia. Schizophrenia bulletin. 1987;13(2):261-76.

7. Guy W. Clinical Global Impressions Scale. ECDEU Assessment Manual for Psychopharmacology US Department of Health, Education and Welfare publication (ADM) 76-338. Rockville, MD: National Institute of Mental Health; 1976. p. 217-22.

8. Endicott J, Spitzer RL, Fleiss JL, Cohen J. The global assessment scale. A procedure for measuring overall severity of psychiatric disturbance. Archives of general psychiatry. 1976;33(6):766-71.

9. Shaffer D, Gould MS, Brasic J, Ambrosini P, Fisher P, Bird H, et al. A children's global assessment scale (CGAS). Archives of general psychiatry. 1983;40(11):1228-31.

10. Young RC, Biggs JT, Ziegler VE, Meyer DA. A rating scale for mania: reliability, validity and sensitivity. The British journal of psychiatry : the journal of mental science. 1978;133:429-35.

11. Montgomery SA, Asberg M. A new depression scale designed to be sensitive to change. The British journal of psychiatry : the journal of mental science. 1979;134:382-9.

12. Tibshirani R. Regression Shrinkage and Selection via the lasso. Journal of the Royal Statistical Society Series B. 1996;58:267–88.

13. Andreasen NC, Carpenter WT, Jr., Kane JM, Lasser RA, Marder SR, Weinberger DR. Remission in schizophrenia: proposed criteria and rationale for consensus. The American journal of psychiatry. 2005;162(3):441-9.

14. Ashburner J, Friston KJ. Unified segmentation. Neuroimage. 2005;26(3):839-51.

15. Del Ser T, Gonzalez-Montalvo JI, Martinez-Espinosa S, Delgado-Villapalos C, Bermejo F. Estimation of premorbid intelligence in Spanish people with the Word Accentuation Test and its application to the diagnosis of dementia. Brain Cogn. 1997;33(3):343-56.

16. Salvador R, Radua J, Canales-Rodriguez EJ, Solanes A, Sarro S, Goikolea JM, et al. Evaluation of machine learning algorithms and structural features for optimal MRI-based diagnostic prediction in psychosis. PloS one. 2017;12(4):e0175683.

17. Canales-Rodriguez EJ, Radua J, Pomarol-Clotet E, Sarro S, Aleman-Gomez Y, Iturria-Medina Y, et al. Statistical analysis of brain tissue images in the wavelet domain: wavelet-based morphometry. Neuroimage. 2013;72:214-26.

18. Radua J, Canales-Rodriguez EJ, Pomarol-Clotet E, Salvador R. Validity of modulation and optimal settings for advanced voxel-based morphometry. Neuroimage. 2014;86:81-90.

19. Landin-Romero R, Amann BL, Sarro S, Guerrero-Pedraza A, Vicens V, Rodriguez-Cano E, et al. Midline Brain Abnormalities Across Psychotic and Mood Disorders. Schizophrenia bulletin. 2016;42(1):229-38.

20. Palau P, Solanes A, Pomarol-Clotet E, Radua J. Beyond only symptoms or only MRI – their combination predicts manic relapses better. Submitted. 2021.

21. Albajes-Eizagirre A, Solanes A, Vieta E, Radua J. Voxel-based meta-analysis via permutation of subject images (PSI): Theory and implementation for SDM. Neuroimage. 2019;186:174-84.
